# MOKA: a pipeline for multiomics bridged SNP-set kernel association test

**DOI:** 10.1093/g3journal/jkaf296

**Published:** 2025-12-19

**Authors:** David Enoma, Dinghao Wang, Ariel Ghislain Kemogne Kamdoum, Rodrigo Ortega Polo, Quan Long, Jingni He

**Affiliations:** Department of Biochemistry and Molecular Biology, Cumming School of Medicine, University of Calgary, Calgary, AB T2N 4N1, Canada; The Mathison Centre for Mental Health Research and Education, Hotchkiss Brain Institute, Cumming School of Medicine, University of Calgary, Calgary, AB T2N 4N1, Canada; Alberta Children's Hospital Research Institute, University of Calgary, Calgary, AB T2N 4N1, Canada; Department of Mathematics and Statistics, Faculty of Science, University of Calgary, Calgary, AB T2N 4N1, Canada; Department of Mathematics and Statistics, Faculty of Science, University of Calgary, Calgary, AB T2N 4N1, Canada; Lethbridge Research and Development Centre, Agriculture and Agri-Food Canada, Lethbridge, AB T1J 4B1, Canada; Department of Biochemistry and Molecular Biology, Cumming School of Medicine, University of Calgary, Calgary, AB T2N 4N1, Canada; The Mathison Centre for Mental Health Research and Education, Hotchkiss Brain Institute, Cumming School of Medicine, University of Calgary, Calgary, AB T2N 4N1, Canada; Alberta Children's Hospital Research Institute, University of Calgary, Calgary, AB T2N 4N1, Canada; Department of Mathematics and Statistics, Faculty of Science, University of Calgary, Calgary, AB T2N 4N1, Canada; Department of Medical Genetics, Cumming School of Medicine, University of Calgary, Calgary, AB T2N 4N1, Canada; Department of Biochemistry and Molecular Biology, Cumming School of Medicine, University of Calgary, Calgary, AB T2N 4N1, Canada; Department of Neuroscience, School of Translational Medicine, Faculty of Medicine, Nursing and Health Sciences, Monash University, Melbourne, VIC 3004, Australia

**Keywords:** bioinformatics, genome-wide association studies, kernel-based association test, functional annotation, post-GWAS analysis, snakemake, workflow management system

## Abstract

The explosion of genomic and multiomics data has created a need for scalable, reproducible tools that integrate functional annotations into genome-wide association studies (GWAS). We introduce the multiomics data bridged Kernel Association test (MOKA) pipeline, a Snakemake-based workflow that automates SNP-set kernel-based association testing by incorporating multiomics data, including gene expression, transcription factor binding, evolutionary conservation scores, and neural network-derived features. This data-bridged architecture enhances variant prioritization and aggregation, improving statistical power in GWAS. MOKA supports population structure correction via spectral decomposition, parallel computation, and post-GWAS analyses, including visualization, Gene Ontology annotation, pathway enrichment, and validation. As a use case, we applied MOKA to a schizophrenia GWAS cohort, identified 89 Bonferroni-significant genes, with a 15.7% validation rate in the disease-specific DisGeNET database and enrichment in pathways relevant to neuropsychiatric disease. MOKA provides a robust, scalable, and extensible framework for functional multiomics integration in genetic studies. It is open-source and available at https://github.com/davidenoma/moka.

## Teaser

Enoma *et al*. present MOKA, an automated Snakemake pipeline that integrates multi-omics annotations such as gene expression, transcription factor binding, evolutionary conservation, and neural network-derived features into SNP-set kernel association tests. MOKA enhances GWAS discovery power and reduces genomic inflation with optional spectral decomposition. Applied to schizophrenia GWAS data, MOKA identified 89 significant genes with improved biological relevance and a 15.7% validation rate in DisGeNET, providing a robust and reproducible framework for post-GWAS analysis.

## Introduction

Genome-wide association studies (GWAS) have been instrumental in identifying genetic variants associated with complex traits and diseases (Wu et al. 2010; Dehghan 2018). However, a large proportion of heritability remains unexplained, particularly due to limitations in detecting the cumulative effects of causal variants (Manolio et al. 2009). Leveraging functional multiomics data has shown promise in improving GWAS by enabling more informed variant selection and aggregation (Wu et al. 2010).

Our previous work introduced kernel-based association tests using a data-bridged architecture (Cao et al. 2021), which integrates external multiomics data to guide variant prioritization and aggregation in downstream analyses. This framework has been successfully applied to diverse modalities, including gene expression (Cao et al. 2022a), brain imaging-derived phenotypes (He et al. 2024 ), transcription factor occupancy (He et al. 2022), and transcription factor binding-informed trans-variants (He et al. 2025). A public database resource was also developed to disseminate genome-wide results (Cao et al. 2022b).

As genomic and multiomics datasets continue to expand in scale and complexity, there is a growing need for robust, scalable, and reproducible tools that streamline processing and analysis. Workflow management systems such as Snakemake (Koster and Rahmann 2018) have gained popularity due to their reproducibility, scalability, and ease of integration with high-performance computing environments. Despite the increasing complexity of the multiomics studies (Cooper and Shendure 2011 ), there is a lack of automated pipelines tailored for kernel-based association tests, a class of statistical methods that model the joint effects of multiple variants in genetic studies. Several related workflows exist. Nf-GWAS pipeline (Song et al. 2021) and Compile GWAS (Hill et al. 2022) provide automated orchestration of conventional GWAS analyses, focusing on quality control, association testing, and result summarization. The Ensembl post-GWAS tool focuses primarily on fine-mapping and credible set identification but does not perform genome-wide testing or integrate functional annotations. Importantly, none of these workflows incorporates external variant-level functional weights or provides end-to-end biological interpretation within a unified framework.

To address these gaps, we introduce the Multiomics Kernel-based Association (MOKA) pipeline, a fully automated analysis workflow built on the Snakemake workflow management system (Koster and Rahmann 2018). MOKA is a modular, reproducible, and scalable framework that integrates externally derived functional weights directly into kernel-based gene-level association tests. This pipeline streamlines the entire analysis process, from multiomics data integration and kernel-based association testing (with correction for population structure) to result visualization, biological annotation, disease database validation, Gene Ontology enrichment, and pathway analysis.

## Methods

### Design of MOKA

The MOKA pipeline is implemented using the Snakemake workflow management system (Koster and Rahmann 2018). Installation requires only a few simple steps, as outlined in the MOKA online documentation (https://github.com/davidenoma/moka).

### Configuration, data and software requirements

MOKA requires input genotype data in PLINK binary format (bed, bim, fam) (Purcell et al. 2007). Configuration is handled through a YAML file (config/config.yaml, see Table 1), which specifies input files, parameters, and auxiliary scripts. To optimize computational efficiency, MOKA supports chromosome-level parallelization by invoking GNU Parallel (Tange 2018) within a single Snakemake rule. Gene regions are defined as ±500 kb from gene boundaries based on the hg38 human genome reference (Mudge et al. 2025). This is a pragmatic choice that captures most proximal regulatory variants. Gene-based GWAS workflows use 1 Mb windows (upstream and downstream) (Barbeira et al. 2018; Kerimov et al. 2021; He et al. 2024); MOKA offers a configurable parameter (default 500 kb) so users can configure those settings and adapt to their needs (Table 1).

**Table 1.** Configuration table for MOKA. yaml format.

Key	Purpose/expected value
genotype_prefix	Basename of the PLINK trio (.bed/.bim/.fam).
weights_type	Short mnemonic for the bridge weights (eg phyloP, VAE-ED).
genotype_file_path	Folder containing the input PLINK files.
weights_file	Path to the CSV of variant weights. Header must include SNP_ID, Chromosome, Position, Weight. sample (weights/weights.csv)
gene_regions_file	CSV listing gene regions (Gene, Start, Stop, Chromosome) that define each SNP set.
disgenet_reference_file	Finally, disgenet_reference_file (“disease_database/{}version.tsv”) that points to a local cache of DisGeNET (Pinero et al. 2017) files.
plink_path	Absolute path to the PLINK executable (leave blank if PLINK is already on $PATH).
is_binary	“TRUE” for case-control phenotypes, “FALSE” for quantitative traits. Guides downstream R scripts.
spectral_decorrelated	“TRUE” to apply transformation based on the spectral decomposition of the genomic relationship matrix (GRM); “FALSE” to run the kernel test without this step.
flank_size	Gene window for MOKA, including upstream and downstream (default is ±500,000).

### Multiomics data bridge

**Fig. 1.**
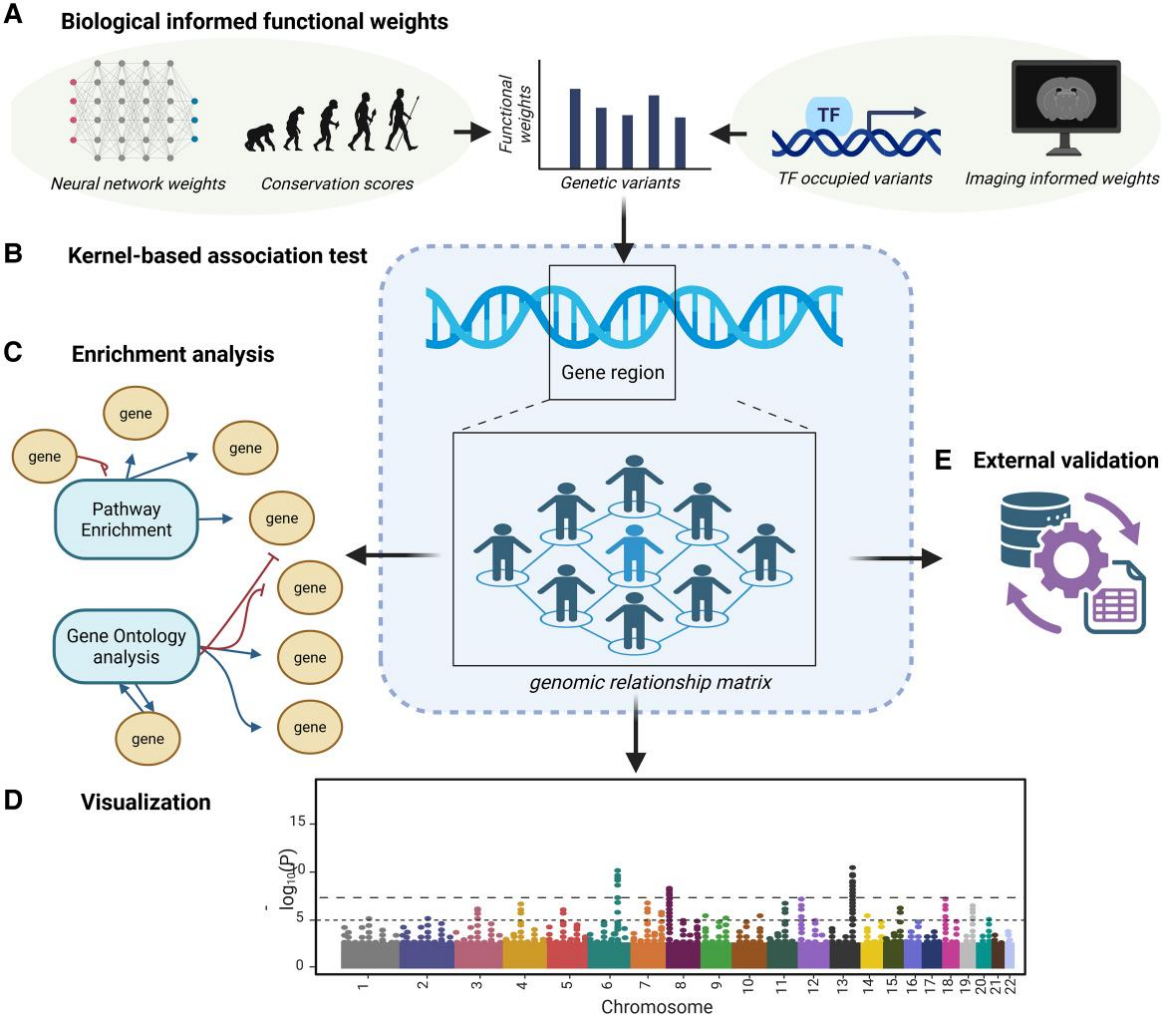
Overview of the MOKA pipeline. a) Biologically informed functional weights derived from diverse multiomics resources, including neural-network predictions, evolutionary conservation scores, transcription-factor occupancy, and brain-imaging–derived annotations, etc., are incorporated as prior information. These variant-level functional weights serve as inputs for the downstream kernel-based association test. b) The MOKA framework integrates genotypes with these functional weights in a kernel-based association model that jointly evaluates the contribution of multiple variants within a gene region while accounting for population structure and relatedness. c) Post-association analyses include KEGG pathway and Gene Ontology (GO) enrichment to identify biological processes and functional themes among significantly associated genes. d) Genome-wide association results are visualized using Manhattan plots to highlight significant loci. e) Significant findings are externally validated against curated knowledge bases such as DisGeNET, quantifying the proportion of associated genes replicated in independent datasets.

Multiomics data sources include user-defined functional genomic annotations at the nucleotide level, capturing biologically relevant features (Fig. 1a), such as cis-regulatory variants, transcription factor binding (He et al. 2022, 2025 ), evolutionary conservation scores (Hubisz and Pollard 2014), gene expression changes (Li et al. 2024), imaging-derived weights, neural network-based (Li et al. 2023) approaches (Enoma et al. 2022), and others that promise to uncover disease-associated variants and genes (Cooper and Shendure 2011). MOKA accepts variant-level functional weights as optional input to incorporate biologically informed priors into the kernel-based association framework. These weights are numerical scores that quantify the presumed functional relevance of individual variants and can be derived from a broad range of multiomics resources, including evolutionary conservation metrics, eQTL effect sizes, neural network-based regulatory predictions, transcription factor occupancy data, or imaging-derived annotations.

The input weight file must be provided in comma-separated format (CSV) with 4 columns (SNP_ID, Chromosome, Position, and Weight) (Table 1), where each row corresponds to a SNP present in the genotype dataset. An example is shown below in Box 1:

Box 1. Evolutionary conservation-derived weights. SNP_ID,Chromosome,Position,Weight

rs885593,10,494221,1.235

rs1581843,10,706253,0.456

rs816306,10,711056,0.457

rs10508203,10,1073263,0.742

… …

Variants not included in the file are automatically assigned a default weight of 1. Weight values are typically continuous between 0 and 1, but MOKA does not impose a specific range, reflecting the relative importance of each SNP.

Currently, MOKA supports a single weight file per analysis run to ensure computational efficiency and interpretability. However, users may conduct multiple analyses using different omics-derived weight sets or combine several sources into a composite weight file prior to analysis. In this way, MOKA provides a flexible and extensible framework for integrating biologically informed priors from diverse omics layers with genome-wide genotype data within a unified kernel-based association model.

### Kernel-based association testing with multiomics weights

Within the input GWAS dataset, each SNP set or gene region is tested using a kernel-based method, which flexibly models epistatic and nonlinear effects of SNPs (Wu et al. 2010). For each individual, a weighted kernel is constructed using the SNPs and the corresponding data-bridged weights (Fig. 1b).


$$K_{w}=\frac{1}{p}XW_{\mathrm{mo}}X^{T}$$

where *X* is the genotype matrix for the SNPs in each gene region, $W_{\mathrm{mo}}$ is a diagonal matrix defined as $W_{\mathrm{mo}}$ = diag $\;({\hat{\beta }}_{1},\ldots ,{\hat{\beta }}_{p}$), with *p* being the number of selected variants. The weights ${\hat{\beta }}_{i}$ are derived from multiomics data integration. This data-bridged weighted kernel ($K_{w}$) is used in the association analysis implemented through a kernel-based test. The test statistic is defined as:


$$Q=Y^{T}K_{w}Y$$

where *Y* is the vector of phenotype values from the GWAS dataset, and $K_{w}$ is the weighted kernel matrix defined above. Under the null hypothesis, *Q* follows a mixture of chi-squared distributions.

The *Q*-score test statistic evaluates whether the SNPs within the gene regions contribute to the observed phenotypic variation. The significance of the association for each gene region is assessed by calculating the *P*-value at a 0.05 threshold (prior to multiple testing correction), based on the null distribution of *Q*.

### Data transformation to control population structure

Uneven genetic relatedness will cause population structure, leading to inflated *P*-values (Kang et al. 2010). Linear mixed models are usually used for controlling this in single-SNP analysis (Lippert et al. 2011). However, here we are carrying out a gene-based set test and aggregating SNPs within the gene region. To handle this problem, we use a transformation based on the spectral decomposition of the genomic relationship matrix (GRM).

Previous work was done for both GWAS (Long et al. 2013) and gene expression analysis (Long et al. 2016) to decorrelate artifacts caused by uneven genetic relatedness using the same decompositions, which we implement as a feature in the MOKA snakemake pipeline (https://github.com/davidenoma/moka).

Assuming the Linear Mixed model without the fixed effect of a SNP takes form:


Y=Zβ+g+ϵ

$$g∼\mathrm{MVN}(0,\;\sigma_{g}^{2}G)$$

$$\epsilon ∼\mathrm{MVN}(0,\;\sigma_{e}^{2}I)$$

where *Y* is the phenotype vector with *n* samples, *Z* is the covariate matrix, and *β* is the vector of fixed effect sizes. *g* represents the random genetic effects, with $\sigma_{g}^{2}$ denoting the genetic variance and *G* being the genomic relationship matrix (GRM). ϵ denotes the residual effects, and $\sigma_{e}^{2}$ is the residual variance.

Then, the variance of Y is given by $\mathrm{Var}(Y)=\;\sigma_{g}^{2\;}G+\sigma_{e}^{2}\;I$, and the unknown parameters ($\sigma_{g}^{2\;}$and $\sigma_{e}^{2}$) can be efficiently estimated using methods such as restricted maximum likelihood (REML) (Lippert et al. 2011; Yang et al. 2011a). Residual structure in *G* can inflate the genome-wide test statistics, a phenomenon usually summarized by the genomic inflation factor $\lambda_{\mathrm{GC}}$, defined as the ratio of the median observed chi-square $(\chi^{2}$) statistic to its null expectation (Devlin and Roeder 1999).

We denote the eigen decomposition of GRM to be $G=USU^{T}$, where *U* is an orthogonal matrix, whose columns are the eigenvectors of *G*, and *S* is a diagonal matrix containing the corresponding eigenvalues.

Substituting this into Var(Y), we obtain:


$$\begin{array}{rl} \mathrm{Var}(Y) & =\;\sigma_{g}^{2\;}G+\sigma_{e}^{2}\;I\\ & =\sigma_{g}^{2\;}\mathrm{US}U^{T}+\;\sigma_{e}^{2}I\\ & =\sigma_{g}^{2\;}USU^{T}+\;\sigma_{e}^{2}UU^{T}\\ & =U(\sigma_{g}^{2\;}S\;+\;\sigma_{e}^{2}I)U^{T} \end{array}$$

Next, we define a transformation matrix $D=(\sigma_{g}^{2\;}S+\sigma_{e}^{2}I{)}^{-\frac{1}{2}}\;U^{T}$.

Subsequently, the transformed varian=Ice of *Y* becomes:


$$\begin{array}{rl} \mathrm{Var}(DY) & =D\;\mathrm{Var}(Y)\;D^{T}\\ & =(\sigma_{g}^{2\;}S+\sigma_{e}^{2}I{)}^{-\frac{1}{2}}\;U^{T}U(\sigma_{g}^{2\;}S+\sigma_{e}^{2}I)U^{T}U(\sigma_{g}^{2\;}S+\sigma_{e}^{2}I{)}^{-\frac{1}{2}}\\ & =(\sigma_{g}^{2\;}S+\sigma_{e}^{2}I{)}^{-\frac{1}{2}}(\sigma_{g}^{2\;}S+\sigma_{e}^{2}I)\;(\sigma_{g}^{2\;}S+\sigma_{e}^{2}I{)}^{-\frac{1}{2}}\\ & =I \end{array}$$

The last step holds because $\left(\sigma_{g}^{2\;}S+\sigma_{e}^{2}I\right)$ is a diagonal matrix.

Now, we apply this transformation to both the phenotype *Y* (with *n* samples) and the genotype *X* (with *n* samples and *p* variants), defining $\tilde{Y}=DY$ and $\tilde{X}=DX$. This transformation ensures that the covariance matrix of $\tilde{Y}$ is the identity matrix, ie $\mathrm{Var}(\tilde{Y})=I$.

So, we apply the MOKA test to the transformed data, we compute the test statistic:


$$Q={\tilde{Y}}^{T}K_{w}\tilde{Y}$$

where $K_{w}=\frac{\tilde{X}W_{\mathrm{mo}}{\tilde{X}}^{T}}{p}\;$ is the kernel matrix and is $W_{\mathrm{mo}}$ a weight matrix that reflects the multiomics weight-specific contribution of each variant and used for MOKA. Spectral decomposition is available as a default functionality in MOKA, which can be toggled on or off in the configuration file of the pipeline (see Table 1).

### Genomic inflation factor ($\lambda_{\mathrm{GC}}$) calculation

Following Devlin and Roeder (1999), we first convert each gene-level *P*-value to its equivalent χ² statistic via the inverse cumulative-distribution function (inverse CDF) of a χ² distribution with one degree of freedom. $\tilde{\chi_{\mathrm{obs}}^{2}}$ denotes the median of these observed statistics. We then divide this value by the theoretical median of a χ² distribution with one degree of freedom, ${\mathrm{CDF}}_{\chi^{2}(1)}^{\text{-}1}(0.5)$:


$$\lambda_{\mathrm{GC}}=\frac{\tilde{\chi_{\mathrm{obs}}^{2}}}{{\mathrm{CDF}}_{\chi^{2}(1)}^{\text{-}1}(0.5)}$$

Because the denominator is approximately by theory, $\lambda_{\mathrm{GC}}$ ≈ 1 indicates well-calibrated test statistics, whereas $\lambda_{\mathrm{GC}}$ > 1.4 signals genomic inflation arising from unmodeled population structure or other confounding effects (Yang et al. 2011b); however, they show that even with perfect population structure matching, $\lambda_{\mathrm{GC}}$ rises roughly in proportion to polygenicity, so gene-level statistics with many contributing variants can have values well above 1 without implying confounding.

### Post-GWAS annotation and gene set enrichment

The output structure is organized into easily navigable folders, including “output_plots” and “result_folder.” KEGG pathway enrichment (snakemake –cores 1 kegg_pathway_analysis) is performed using *PathfindR* (Ulgen et al. 2019) package, and the Gene Ontology analysis (snakemake –cores 1 go_analysis) is performed with *g:Profiler* (Reimand et al. 2007) package (Fig. 1c).

DisGeNET (Pinero et al. 2017) is a database encompassing 1,134,942 gene-disease associations (GDAs) involving 21,671 genes and 30,170 traits. DisGeNET provides specific summaries for each disease within this platform, detailing gene associations and information on identified significant genes after multiple testing corrections and the proportions of associations are in the database (Fig. 1e) (snakemake –cores 1 disgenet_annotation_005).

## Results

### Configuration of MOKA and multiomics data bridge

In this demonstration, we used 17-way human and primates accelerated conservation scoring (Siepel et al. 2005; Pollard et al. 2010) as weights ($W_{\mathrm{mo}}$) for each variant. The acceleration measures quantify the extent of human-specific sequence change, such as sites that have diverged more rapidly than expected under neutrality. Negative phyloP (Pollard et al. 2010) scores already encode acceleration as −log₁₀*p* from a likelihood-ratio test, so negative values (eg phyloP ≤ −3, corresponding to *P* ≤ 10⁻³) signal human-specific evolution and associated disorder. These annotations are of particular interest in schizophrenia, a human-complex disease (Doan et al. 2016; Levchenko et al. 2018), providing a biologically informed basis for SNP aggregation.

In the neural-network weights’ data bridge, weights ($W_{\mathrm{mo}}$) were obtained by taking the element-wise sum of the variant-specific encoder and decoder weight matrices from a variational auto-encoder (VAE) (Kingma and Welling 2013) model, as previously described by Enoma. The network was trained on the input genotype data, so after tuning and convergence, the neural network weights learnt in the training process of the latent representations for reconstruction are extracted (Unterthiner et al. 2020; Herrmann et al. 2024).

### Association results

**Fig. 2.**
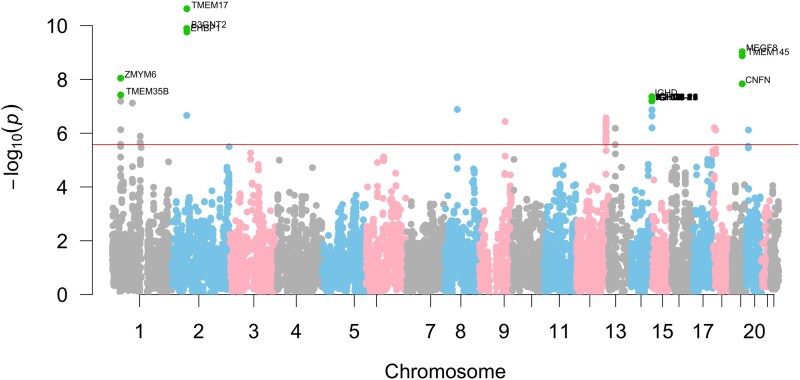
Example manhattan plot of association results.

Figure 2 presents a Manhattan plot of association results using negative conservation score–based weights, with genes on chromosomes 1 to 22 plotted against –log₁₀. The Bonferroni-corrected significance threshold (*P* = 2.64e−06) is shown as a horizontal line. Using human accelerated conservation scores as weights, 89 genes were significantly associated with schizophrenia. The top-associated gene, TMEM17, encodes a transmembrane protein involved in ciliogenesis and neural signaling, and has previously been implicated in schizophrenia (Bigdeli et al. 2021).

### External database validation

For validation, we used DisGeNET disease-specific database as a reference database (schizophrenia in this case). The validation proportion was calculated as the fraction of false discovery rate (FDR)-significant genes at *P*- < 0.05 (Benjamini and Hochberg 1995), that overlapped with DisGeNET entries, ie the number of significant genes found in DisGeNET divided by the total number of significant genes identified in the analysis.

Applying the MOKA association test to the schizophrenia GWAS dataset identified 2,245 significant genes, of which 15.7% (*n* = 351) were also reported in the DisGeNET database. To benchmark performance, we compared these results with 2 established association methods: SKAT (Wu et al. 2010), a kernel-based test aggregating variant effects across genomic regions; and REGENIE (Mbatchou et al. 2021), a scalable whole-genome regression framework designed for large-scale association studies. SKAT identified 2,530 genes, of which 13.3% (*n* = 337) overlapped with DisGeNET (genomic-inflation factor of $\lambda_{\mathrm{GC}}$ ≈ 113), and REGENIE identified 958 genes, with 12.0% (*n* = 115) supported by DisGeNET. These overlap proportions represent the fraction of genes detected by each method that were independently annotated in DisGeNET as schizophrenia-associated, providing an external indicator of the biological plausibility and validity of the detected associations.

### Spectral transformation controls genomic inflation factor

The MOKA pipeline incorporates a transformation based on the spectral decomposition of the variance component to correct for test statistic inflation in kernel-based association analyses. This procedure improves calibration while preserving the biological relevance conveyed by functional variant weights (see Method). We define several modes of operation for clarity. MOKA-Ph refers to a MOKA run using phyloP evolutionary conservation scores as per-SNP functional weights, whereas MOKA-ED uses encoder–decoder (VAE)–derived weights (Enoma et al. 2025). The “-COR” suffix indicates that spectral correction is applied to the genomic relationship matrix; runs without “-COR” omit this step. These options are specified in the configuration file (config.yaml) by setting the appropriate weight file and toggling the spectral_correction parameter (true/false).

In our schizophrenia GWAS, SKAT (Wu et al. 2010) yielded a highly inflated genomic inflation factor of $\lambda_{\mathrm{GC}}$ ≈ 113, indicating substantial confounding and false positives. REGENIE's whole-genome mixed model reduced this to ≈ 4.7, but lingering stratification remains. Using phyloP acceleration scores in MOKA (MOKA-Ph) reduced SKAT's inflation to 10.5. Applying MOKA's spectral correction (MOKA-Ph-COR) further lowered $\lambda_{\mathrm{GC}}$ to ≈ 4.3. While still above the ideal benchmark of <1.2, this matches REGENIE's calibration while retaining mechanistically informed weighting for improved power.

The cohort-wide comparison (Table 2) shows SKAT inflates the test statistics ($\lambda_{\mathrm{GC}}$ ≈ 6.9 to 112.8), while REGENIE reduced this to 3.3 to 4.7. Incorporating encoder–decoder VAE weights in MOKA (MOKA-ED) reduces the inflation to 4.0 to 10.1, but remains above REGENIE. The decisive improvement comes from the spectral decomposition of the GRM: MOKA-ED-COR further reduces inflation to 2.5 to 4.6. In schizophrenia, $\lambda_{\mathrm{GC}}$ drops from 113 (SKAT) to 10.1 (MOKA-ED) and 4.6 (MOKA-ED-COR), comparable to REGENIE. These findings demonstrate that MOKA with spectral correction yields well-calibrated, biologically informed association tests, although additional adjustment for population structure may still be beneficial.

**Table 2.** Genomic-inflation factor ($\lambda_{\mathrm{GC}}$) across 5 cohorts for 4 association methods (REGENIE (Mbatchou et al. 2021); SKAT (Wu et al. 2010); MOKA-ED = MOKA with encoder–decoder neural-network weights; MOKA-ED-COR MOKA-ED with spectral (GRM) decomposition).

Cohort	REGENIE	SKAT	MOKA-ED	MOKA-ED-COR
Chron's disease	3.455	7.747	4.247	2.452
Coronary arterial disease	3.246	6.882	3.981	2.513
Hypertension	3.263	7.512	4.192	2.527
Bipolar disorder	3.608	11.234	4.921	2.623
Schizophrenia	4.718	112.766	10.085	4.627

### Gene ontology analysis

**Fig. 3.**
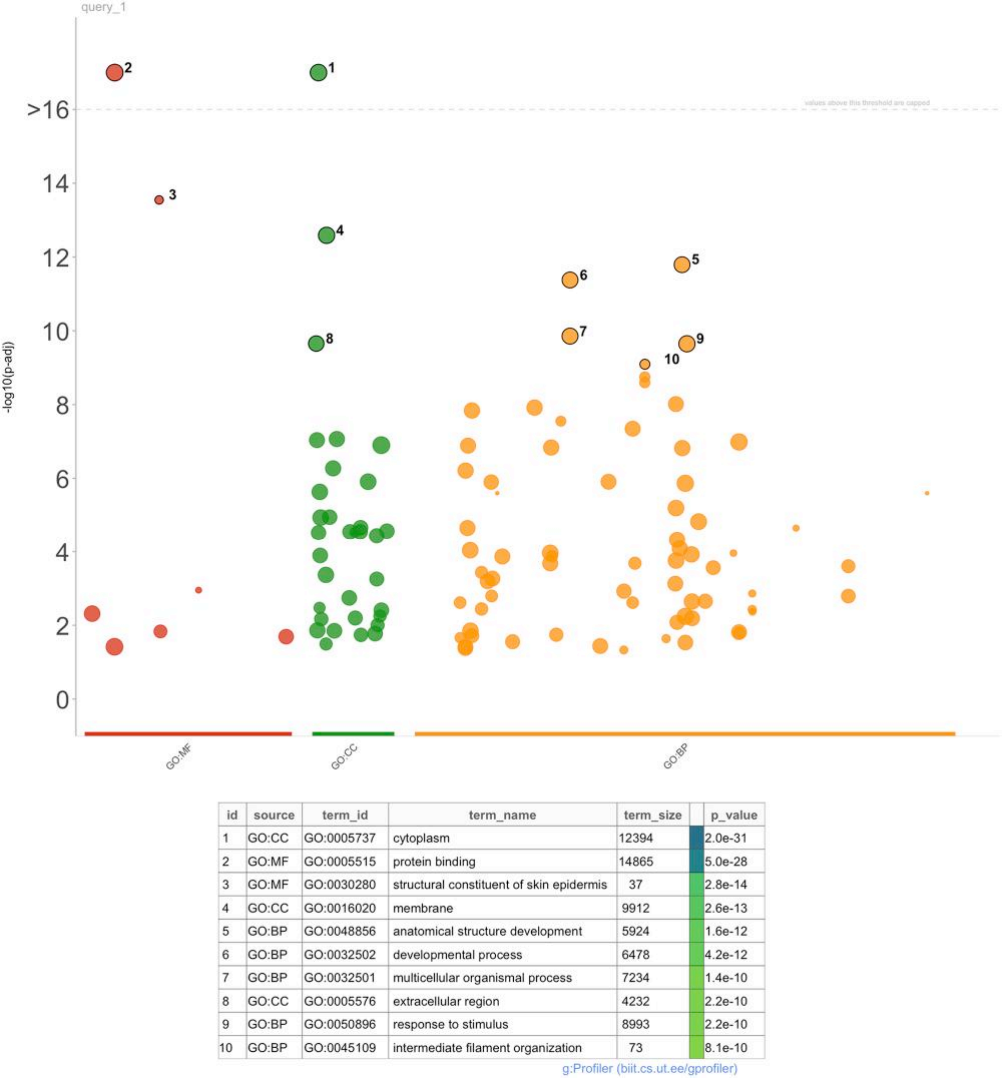
Gene ontology (GO) enrichment analysis of significant genes identified by MOKA. Each dot represents a significantly enriched GO term among genes identified in the MOKA association analysis. The 3 GO domains are shown along the *x*-axis: molecular function (GO:MF, red), cellular component (GO:CC, green), and biological process (GO:BP, orange). The *y*-axis shows the enrichment significance as –log₁₀(adjusted *P*), with higher values indicating stronger enrichment. Dot size corresponds to the number of genes annotated to each GO term, and the top-ranked terms are labeled with their IDs and names. The table below lists the top ten most significant GO terms, including their GO identifiers, term names, total term size, and adjusted *P*-values.

Gene ontology analysis in MOKA (Fig. 3) of FDR-significant results (*P* < 0.05) identified top terms for cellular component (cytoplasm, *P* = 2.0e−31), molecular function (protein binding, *P* = 5.0e−28), and biological process (anatomical structure development, *P* = 1.6e−12) for schizophrenia, respectively.

### KEGG pathway enrichment

**Fig. 4.**
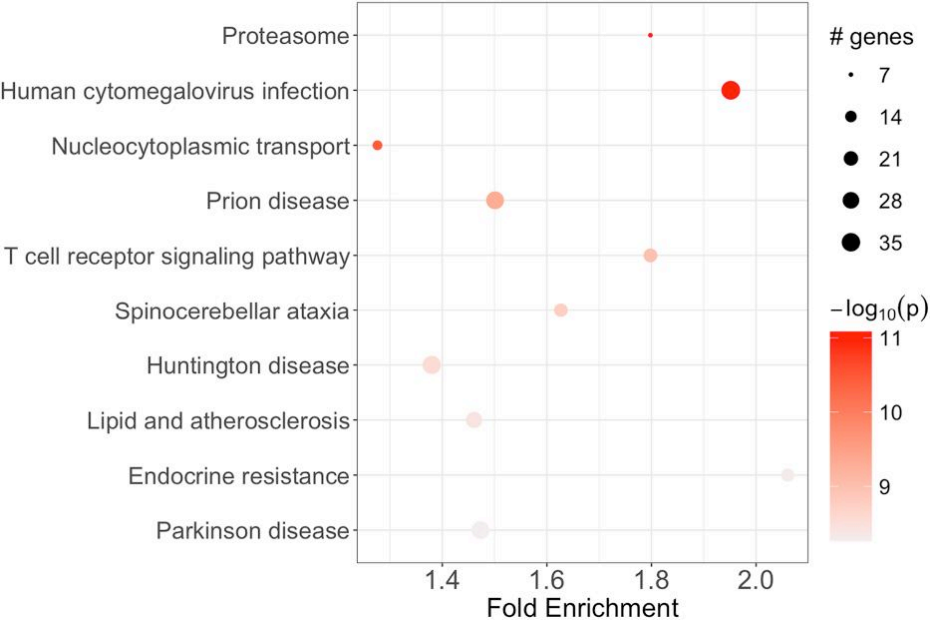
KEGG pathway enrichment analysis of MOKA-identified genes. Each dot represents a significantly enriched KEGG pathway based on genes identified by the MOKA association test. The *x*-axis denotes fold enrichment (ratio of observed to expected gene counts), and the *y*-axis lists pathway names. Dot size indicates the number of genes associated with each pathway, and the color gradient reflects statistical significance (−log₁₀(*P*)). Darker colors indicate higher significance levels. Pathways surpassing the FDR-adjusted *P* < 0.05 threshold are shown.

KEGG pathway enrichment analysis in MOKA (Fig. 4) of FDR-significant results (*P* < 0.05) identified several significantly enriched pathways. The top 5 pathways were Proteasome (*P* = 1.0e−11), human cytomegalovirus infection (*P* = 2.0e−10), nucleocytoplasmic transport (*P* = 4.0e−9), prion disease (*P* = 8.0e−9), and T-cell receptor signaling pathway (*P* = 1.2e−8).

## Conclusion

MOKA is a scalable, automated Snakemake pipeline that enhances GWAS by integrating multiomics data through SNP-set kernel association tests and applying spectral decomposition to control genomic inflation and population structure. It supports comprehensive post-GWAS analyses and visualization.

In the schizophrenia GWAS, SKAT produced extreme inflation ($\lambda_{\mathrm{GC}}$ ≈ 113) with a validation proportion of 13.3%. MOKA, using negative phyloP conservation scores as variant-level weights, reduced $\lambda_{\mathrm{GC}}$ to 10.5 and improved validation to 15.7%. Activating the spectral decorrelation step, which applies a spectral decomposition of the genomic relationship matrix to correct for population structure and test statistic inflation, further reduced inflation to 4.3. Across WTCCC and schizophrenia datasets, SKAT yielded $\lambda_{\mathrm{GC}}$ values between 6.9 and 113, while MOKA with correction reduced this to 2.5 to 4.6, comparable to REGENIE. Liu et al.(Liu et al. 2013) demonstrated that gene-based tests are susceptible to inflation from gene length and allele-frequency heterogeneity, and that single-SNP genomic control can be overly conservative. MOKA addresses these challenges by providing a robust, flexible, and user-friendly framework for multiomics integration and kernel-based association analysis, accessible to the broader research community.

## Data Availability

The genotype datasets are from the Molecular Genetics of Schizophrenia—nonGAIN Sample (MGS_nonGAIN) (dbGaP Study Accession: phs000167.v1.p1) are available at https://www.ncbi.nlm.nih.gov/projects/gap/cgi-bin/study.cgi?study_id=phs000167.v1.p1 and Wellcome Trust Case Control Consortium (WTCCC GWAS) (Wellcome Trust Case Control 2007) are in the public domain. The MOKA Pipeline is freely available on GitHub under the MIT license at https://github.com/davidenoma/moka. The official Docker image is provided at: https://hub.docker.com/r/davidenoma/moka-gwas. We have provided a test genotype dataset (genotype_data/) along with corresponding phyloP conservation scores (test_geno_weights) to facilitate software testing and reproducibility. The test dataset comprises randomly simulated genotypes containing 100 SNPs per chromosome across all 22 chromosomes, with simulated phenotypes for 1,000 cases and 1,000 controls. This example dataset allows users to verify installation, confirm correct execution of the pipeline, and explore the input and output structure of MOKA. The Conjoint Health Research Ethics Board (CHREB) at the University of Calgary approved this work with ID REB23-0045_REN. The PhyloP (Pollard et al. 2010) conservation scores for each variant position may be downloaded from the UCSC genome browser(Nassar et al. 2023).
